# Supplementary material for: Toll-like Receptor 2 Is Associated with the Immune Response, Apoptosis, and Angiogenesis in the Mammary Glands of Dairy Cows with Clinical Mastitis
Source: Int J Mol Sci. 2022 Sep 14;23(18):10717. doi: 10.3390/ijms231810717 (PMC9504312; doi:10.3390/ijms231810717)

The results of power calculation

The sample size of control group (C) and experimental group (CM) was 3 (n=3), we set type I error α=0.05 for hypothesis testing.According to the formula：

$$z_{1-\beta} = \sqrt{\frac{2n_1n_2(mean_1-mean_2)^2}{(sd_1^2+sd_2^2)(n_1+n_2)}} - z_{1-\alpha/2}$$

The type II error β and power of this study were calculated.

	Group	Mean	SD	β (Ⅱ error)	**Power**
TLR2 qPCR (Fig. 4 F)	C	1.1255	0.08143	0.085	**91.5%**
	CM	2.85629	0.89547		
TLR2 WB (Fig. 4 G)	C	0.82922	0.11126	0.001	**99.9%**
	CM	1.91981	0.34288		
CASP8 qPCR (Fig. 6 H)	C	0.95025	0.04859	0	**100.0%**
	CM	2.50618	0.28414		
CASP8 WB (Fig. 6 J)	C	1.44254	0.37762	0	**100.0%**
	CM	2.69063	0.11917		
Tie2 qPCR (Fig. 6 I)	C	0.58929	0.29573	0.115	**88.5%**
	CM	0.04961	0.00748		
Tie2 WB (Fig. 6 J)	C	1.03052	0.0359	0	**100.0%**
	CM	0.65368	0.03905		

Based on preliminary studies (mean ± sd in group C and group CM), a post-hoc power calculations was performed. A sample of 6 patients (3 in group C; 3 in group CM) achieves **91.5%**, **99.9%**, **100%**, **100%**, **88.5%**, **100%** power to detect a difference in means between groups at a two-sided alpha of **0.05**, respectively. All the power＞85%, Therefore, the sample size of the study was reasonable.

non-parametric test (Wilcoxon rank-sum test)

TLR2 qPCR (Fig. 4 F)					
	Group Median(rank)		Wilcoxon rank-sum test	Wilcoxon rank-sum test	*p*
	C (n=3)	CM (n=3)	***U*** value	***z*** value	
Expression	1.182(1.0,1.2)	2.424(2.0,4.1)	0.000	-1.964	0.04953*

* *p* <0.05 ** *p* <0.01

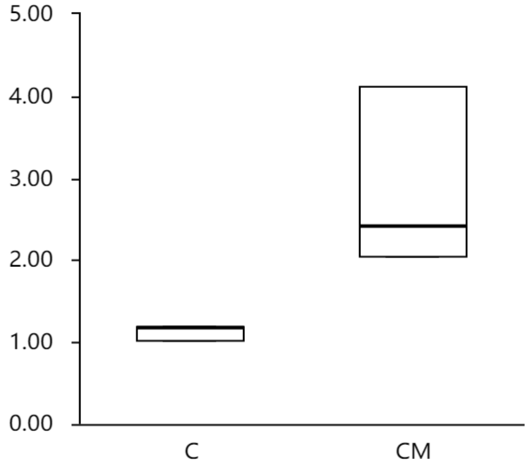

TLR2 WB (Fig. 4 G)					
	Group Median(rank)		MannWhitney test statistic ***U*** value	MannWhitney test statistic ***z*** value	*p*
	C (n=3)	CM (n=3)			
Expression	0.842(0.7,1.0)	1.707(1.6,2.4)	0.000	-1.964	0.04953*

* *p* <0.05 ** *p* <0.01

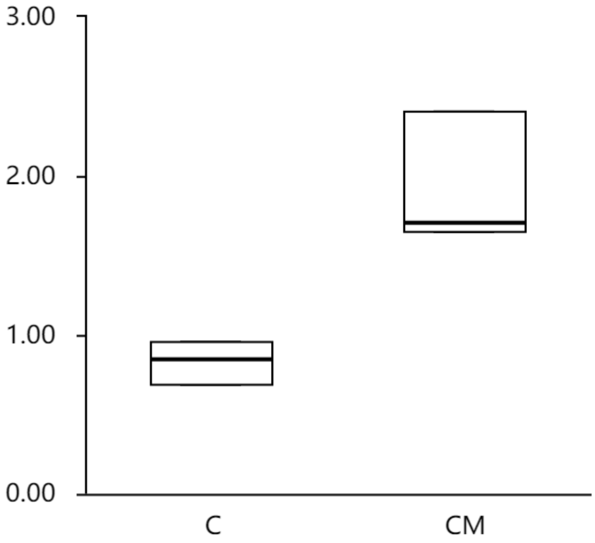

CASP8 qCPR (Fig. 6 H)

	Group Median(rank)		MannWhitney test statistic ***U*** value	MannWhitney test statistic ***z*** value	*p*
	C (n=3)	CM (n=3)			
Expression	0.964(0.9,1.0)	2.368(2.2,2.9)	0.000	-1.964	0.04953*

* *p* <0.05 ** *p* <0.01

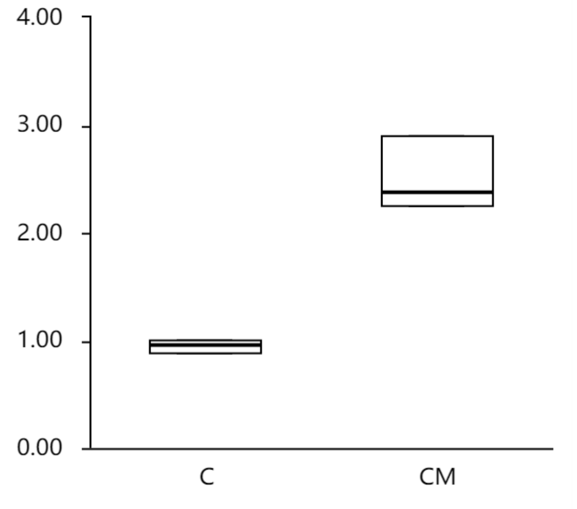

CASP8 WB (Fig. 6 J)

	Group Median(rank)		MannWhitney test statistic ***U*** value	MannWhitney test statistic ***z*** value	*p*
	C (n=3)	CM (n=3)			
Expression	1.405(1.0,1.9)	2.736(2.5,2.8)	0.000	-1.964	0.04953*

* *p* <0.05 ** *p* <0.01

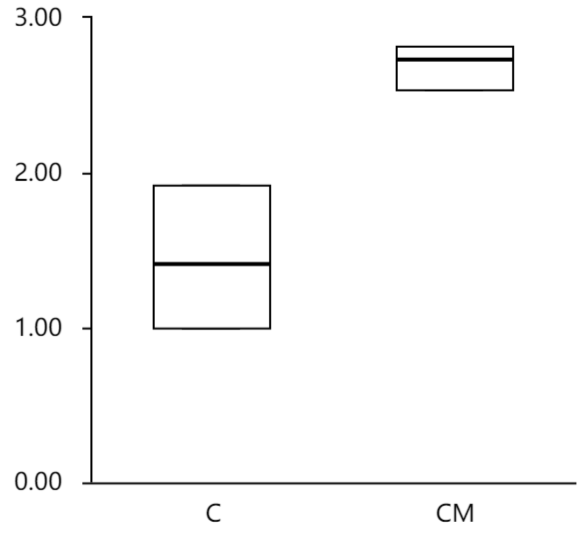

Tie2 qPCR (Fig. 6 I)					
	Group Median(rank)		MannWhitney test statistic ***U*** value	MannWhitney test statistic ***z*** value	*p*
	C (n=3)	CM (n=3)			
Expression	0.436(0.3,1.0)	0.051(0.0,0.1)	0.000	-1.964	0.04953*

* *p* <0.05 ** *p* <0.01

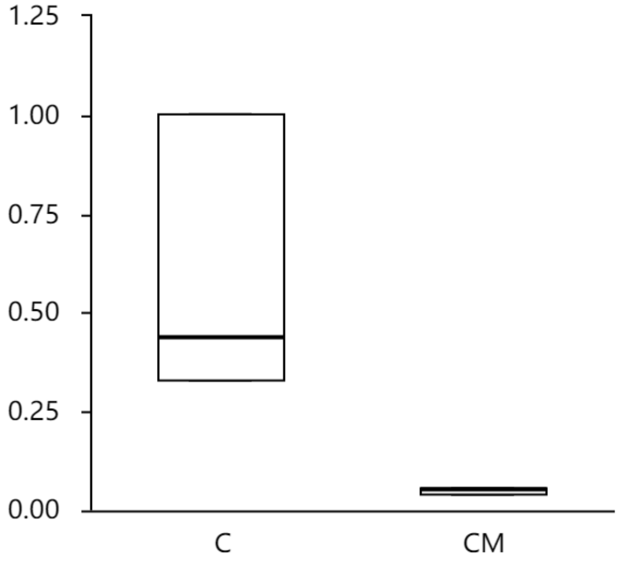

Tie2 WB (Fig. 6 J)					
	Group Median(rank)		MannWhitney test statistic ***U*** value	MannWhitney test statistic ***z*** value	*p*
	C (n=3)	CM (n=3)			
Expression	1.011(1.0,1.1)	0.650(0.6,0.7)	0.000	-1.964	0.04953*

* *p* <0.05 ** *p* <0.01

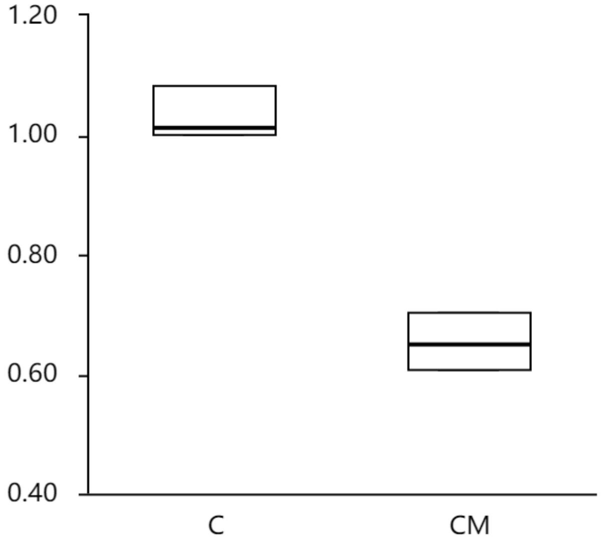

Alevoli (Fig. 6 F)

	Group Median(rank)		MannWhitney test statistic ***U*** value	MannWhitney test statistic ***z*** value	*p*
	C (n=8)	CM (n=8)			
Expression	7.250(5.1,14.0)	48.940(34.5,61.0)	0.000	-3.337	0.00077**

* *p* <0.05 ** *p* <0.01

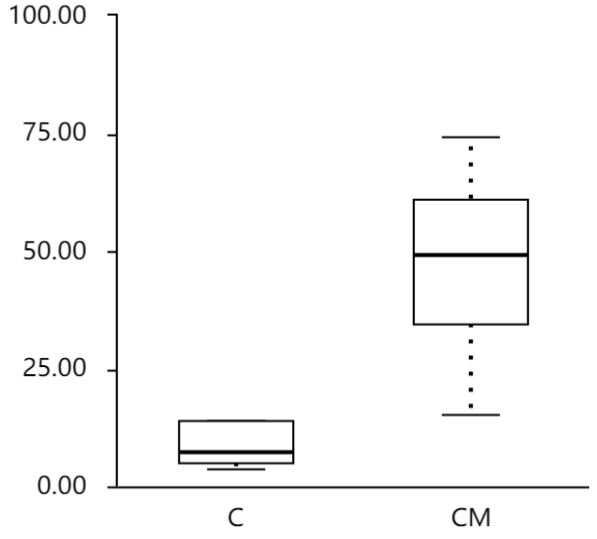

Blood vessel (Fig. 6 G)

	Group Median(rank)		MannWhitney test statistic ***U*** value	MannWhitney test statistic ***z*** value	*p*
	C (n=8)	CM (n=8)			
Expression	5.000(4.6,8.6)	43.330(38.4,56.4)	0.000	-3.132	0.00075**

* *p* <0.05 ** *p* <0.01

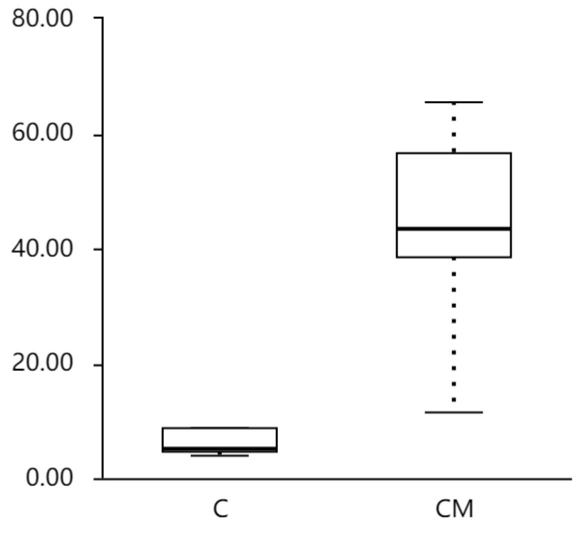

Supplement: Supplementary file 1 [file ijms-23-10717-s001.zip › Power and non-parametric test.pdf]
