# Supplementary figures and images for: Toll-like Receptor 2 Is Associated with the Immune Response, Apoptosis, and Angiogenesis in the Mammary Glands of Dairy Cows with Clinical Mastitis
Source: Int J Mol Sci. 2022 Sep 14;23(18):10717. doi: 10.3390/ijms231810717 (PMC9504312; doi:10.3390/ijms231810717)

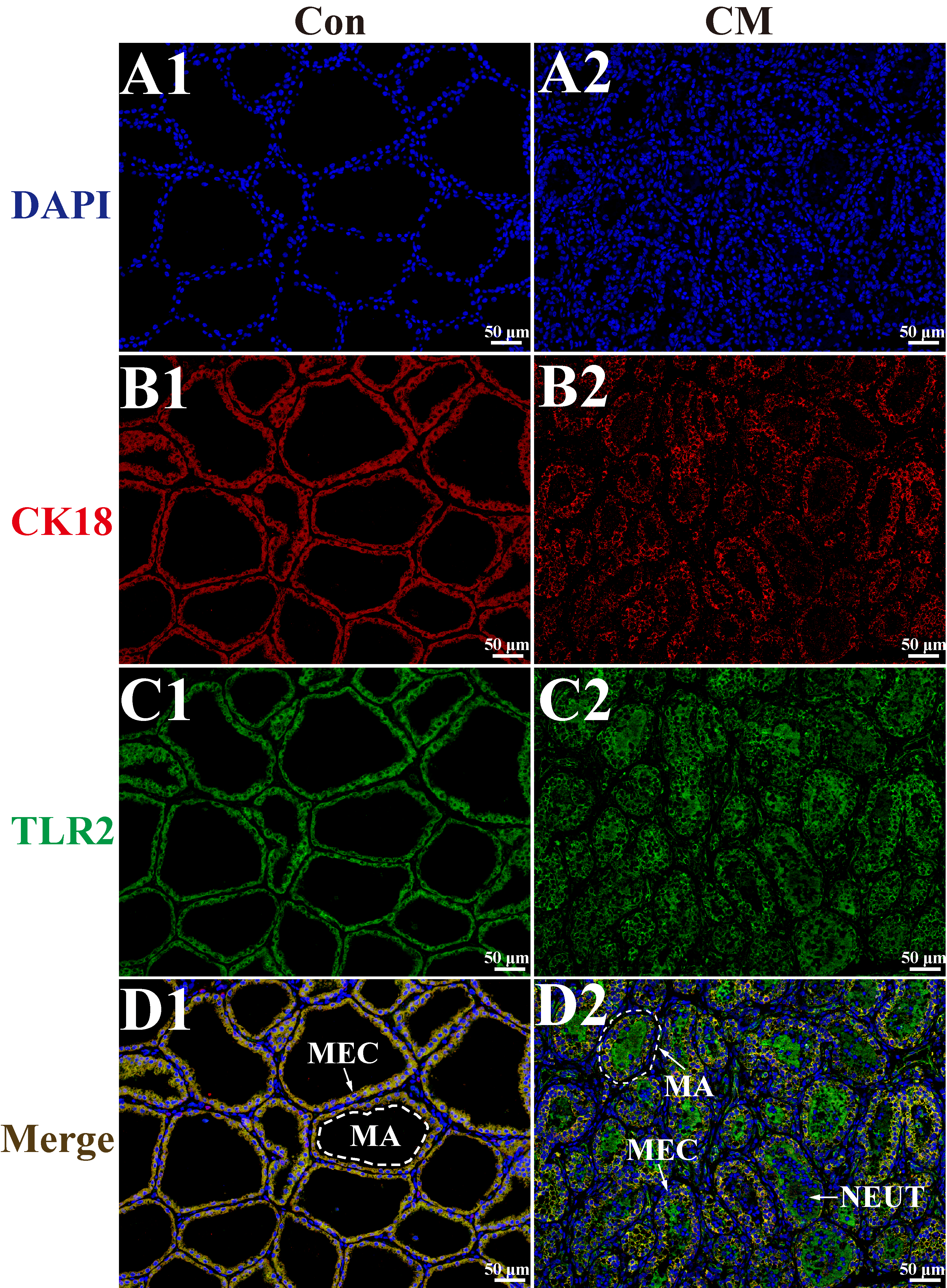

Supplement: Supplementary file 1 [file ijms-23-10717-s001.zip › Figure S1.tif]

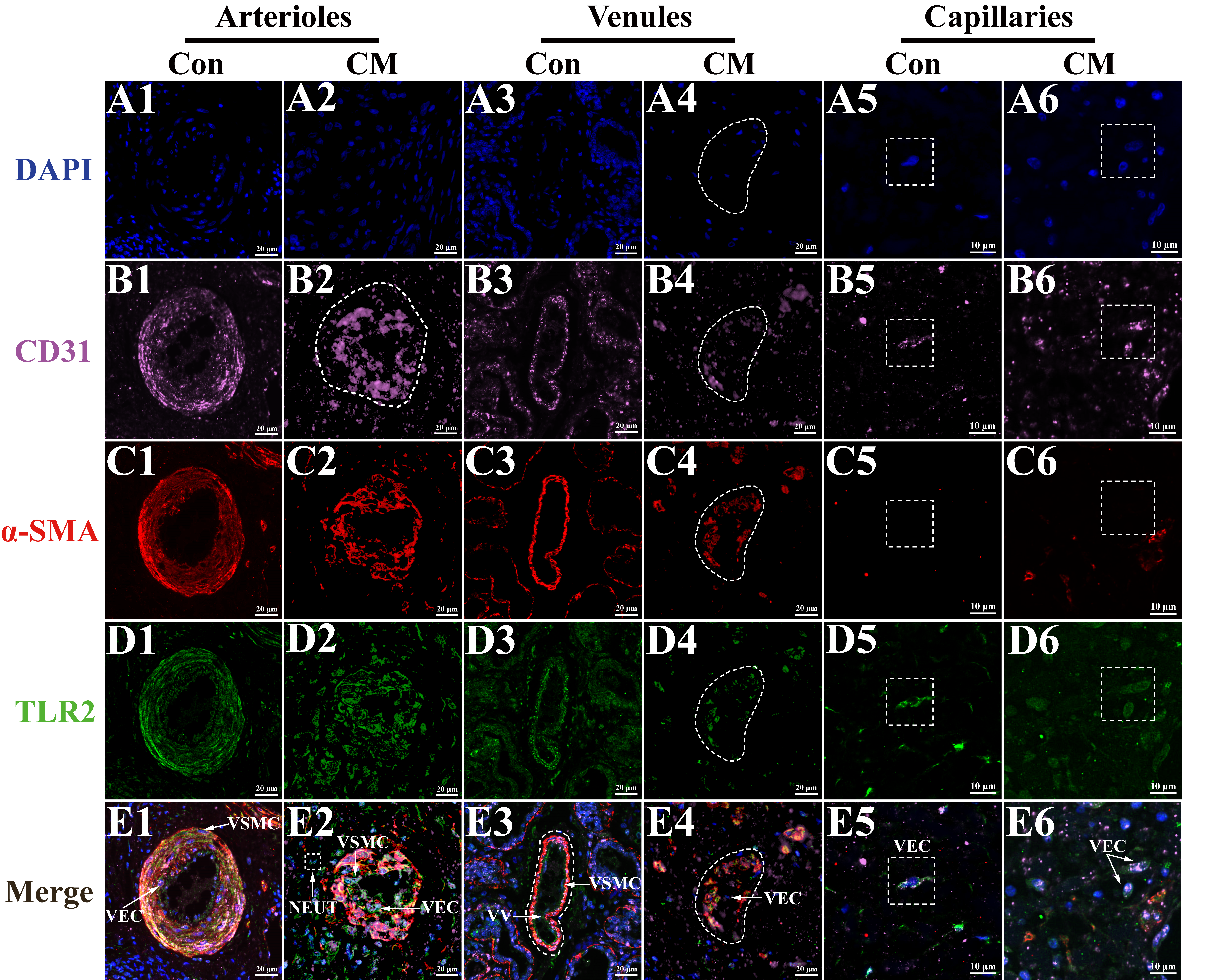

Supplement: Supplementary file 1 [file ijms-23-10717-s001.zip › Figure S2.tif]

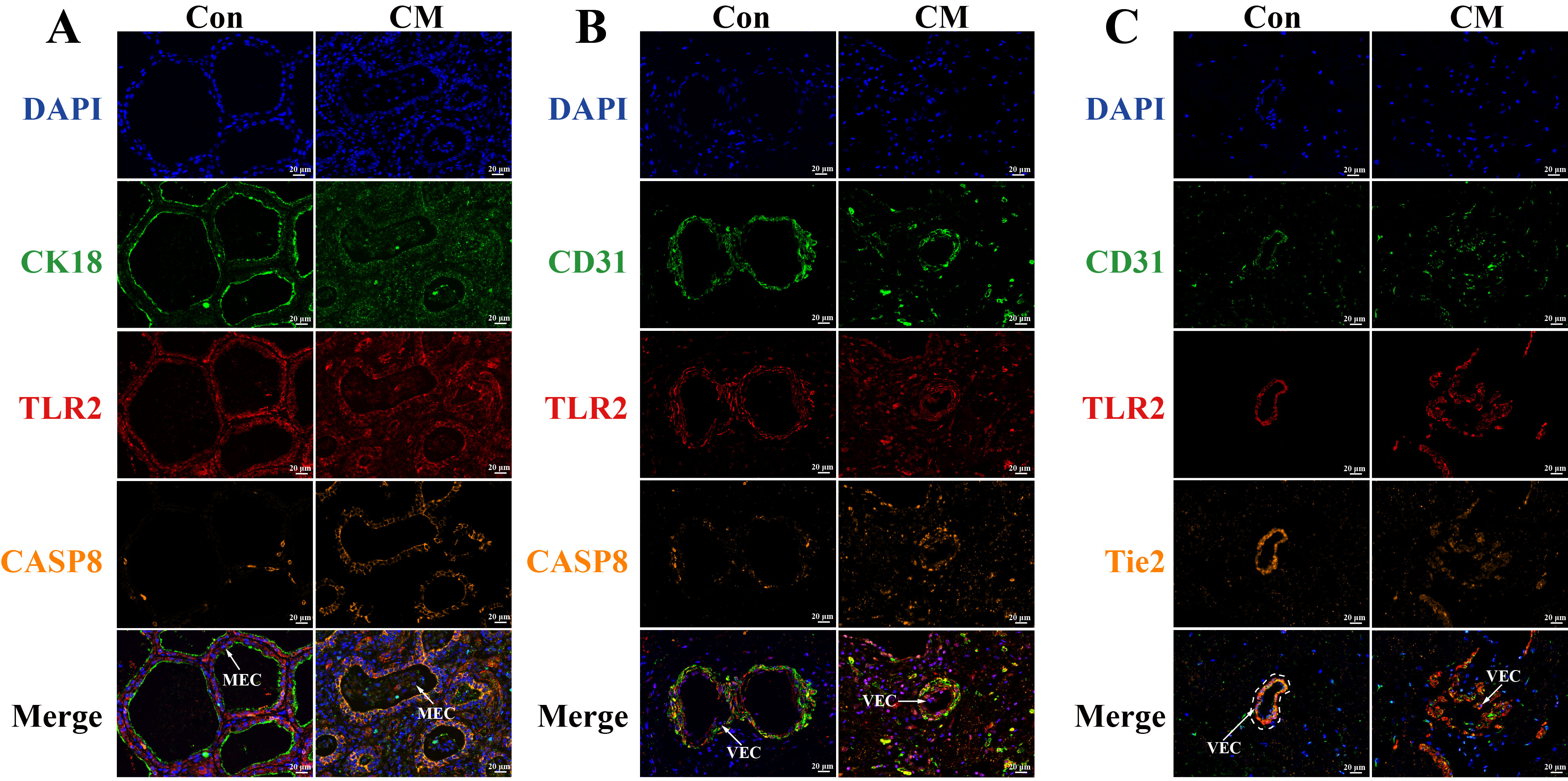

Supplement: Supplementary file 1 [file ijms-23-10717-s001.zip › Figure S3.tif]

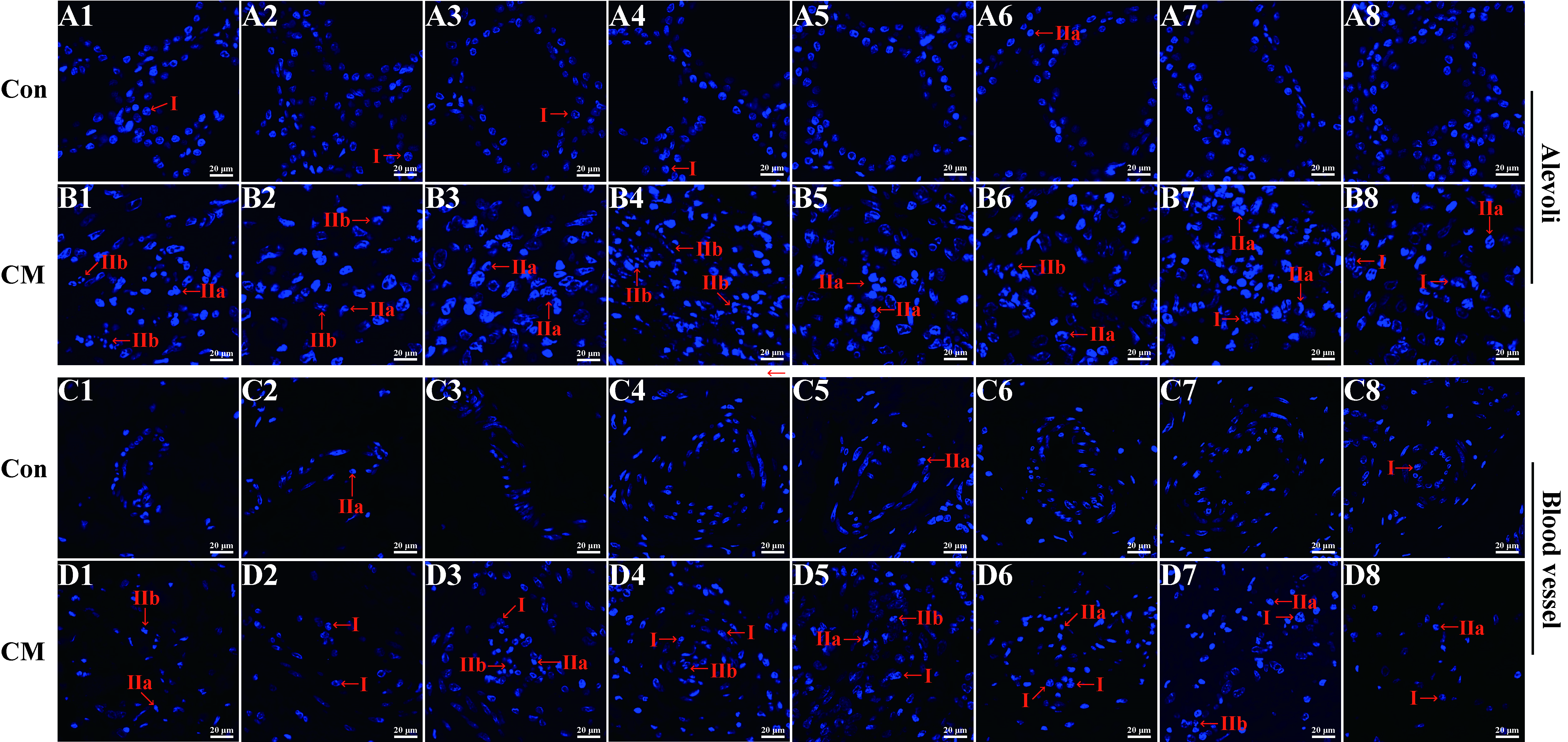

Supplement: Supplementary file 1 [file ijms-23-10717-s001.zip › Figure S4.tif]

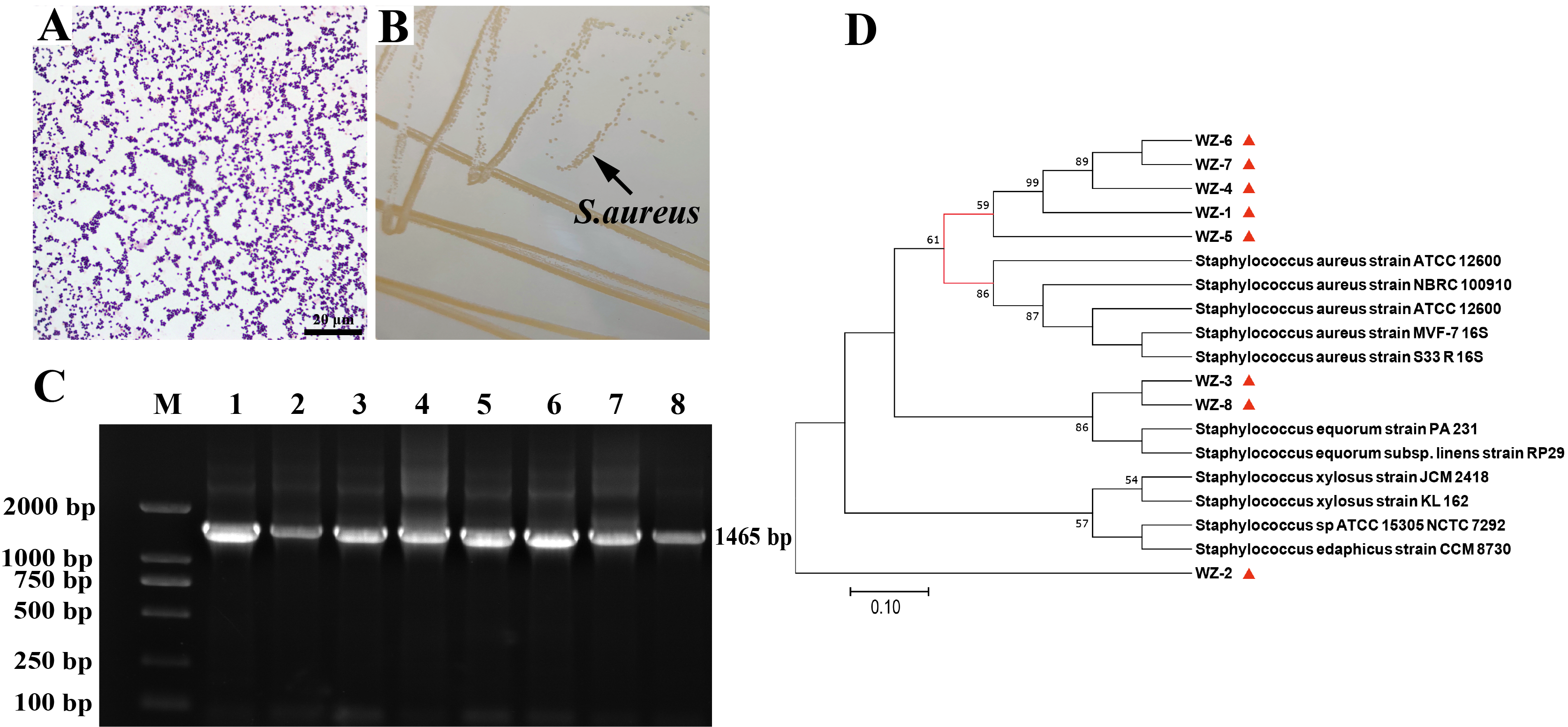

Supplement: Supplementary file 1 [file ijms-23-10717-s001.zip › Figure S5.tif]
